# Hepatitis C Virus Infection: Molecular Pathways to Steatosis, Insulin Resistance and Oxidative Stress

**DOI:** 10.3390/v1020126

**Published:** 2009-08-11

**Authors:** Sophie Clément, Stéphanie Pascarella, Francesco Negro

**Affiliations:** 1 Division of Clinical Pathology, University Hospitals, Geneva, Switzerland; E-Mails: Sophie.Clement@unige.ch (S.C.); Stephanie.Pascarella@unige.ch (S.P.); 2 Division of Gastroenterology and Hepatology, University Hospitals, Geneva, Switzerland

**Keywords:** hepatitis C, reactive oxygen species, insulin signaling, lipid accumulation

## Abstract

The persistent infection with hepatitis C virus is a major cause of chronic liver disease worldwide. However, the morbidity associated with hepatitis C virus widely varies and depends on several host-related cofactors, such as age, gender, alcohol consumption, body weight, and co-infections. The objective of this review is to discuss three of these cofactors: steatosis, insulin resistance and oxidative stress. Although all may occur independently of HCV, a direct role of HCV infection in their pathogenesis has been reported. This review summarizes the current understanding and potential molecular pathways by which HCV contributes to their development.

## 1. Introduction

**Figure 1.**
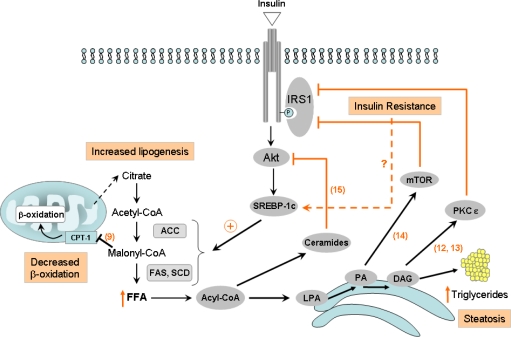
Diagram connecting the insulin pathway and fatty acid (FA) biosynthesis. Insulin resistance may lead to steatosis by inducing the expression and/or the maturation of sterol regulatory element binding protein (SREBP)-1, leading to the increased expression of the enzyme acetyl-CoA carboxylase (ACC) and FA synthase (FAS). Insulin also inhibits FA β-oxidation by increasing malonyl-CoA, a potent inhibitor of carnitine palmitoyltransferase type 1 (CPT)-1, responsible for FA mitochondrial import. Conversely, intermediates in the triglyceride synthesis pathway may induce insulin resistance by activating inhibitors of insulin signaling, including protein kinase C (PKC)-ɛ, by phosphatidic acid (PA), mammalian target of rapamycin (mTOR) by diacyglycerol (DAG). Ceramides can inhibit Akt-mediated insulin signaling. FFA: free fatty acid, LPA: lysophosphatidic acid. Numbers refer to the bibliographic references.

Hepatitis C virus (HCV) is a member of the *Flaviviridae* family responsible for acute and chronic liver disease [1]. Infection with HCV is common, with an average worldwide prevalence of 3% [2]. Acute HCV infection becomes persistent in about 85% of cases [3] and may cause chronic hepatitis leading to cirrhosis and, eventually, hepatocellular carcinoma (HCC) [4]. HCV-induced end-stage liver disease is currently the leading indication to liver transplant in most Western countries. HCV induces several complex pathways leading to insulin resistance (IR), steatosis, fibrosis, inflammation, apoptosis, and HCC [5–8]. With emerging insight into the pathogenic mechanism leading to liver failure, oxidative stress, steatosis and IR are now proposed as important initiators of HCV pathogenesis and are believed to be closely interconnected. Although the link between oxidative stress, steatosis and IR is complex, and the exact sequence of events is unclear, different extrahepatic (for instance adipose tissue or diet) as well as intrahepatic mechanisms have been suggested to explain this interconnection in a more general context. Indeed, IR may lead to steatosis and conversely, steatosis (or more exactly, the accumulation of fatty acid (FA) derivatives used for triglyceride synthesis) can induce IR. Figure 1 gives an overview of some described intrahepatic mechanisms.

First, it has been reported that *de novo* FA synthesis, i.e. lipogenesis (a pathway which is stimulated by insulin) is increased in the case of IR and not decreased as one would expect. Indeed, paradoxically, IR contributes to the activation of lipogenesis through the activation of both the expression and post-translational maturation of sterol regulatory element binding protein (SREBP)-1c, a transcription factor leading to the increased expression of several lipogenic enzymes including acetyl-CoA carboxylase (ACC) and FA synthase (FAS). Moreover, insulin inhibits FA β-oxidation by increasing the level of malonyl-CoA, a potent inhibitor of carnitine palmitoyltransferase (CPT)-1 responsible for FA mitochondrial import [9]. Conversely, several lines of evidence suggest that lipid metabolites can induce IR. Indeed, while triglycerides themselves have been shown to be nontoxic *per se* [10, 11], intermediates of the triglyceride biosynthesis pathway can activate inhibitors of insulin signaling: excess diacyglycerol (DAG) activates protein kinase C (PKC)-ɛ, which phosphorylates insulin receptor substrate (IRS)-1 on serine residues, thereby leading to the decrease of phosphatidylinositol 3 (PI3)-kinase activation [12, 13]; phosphatidic acid (PA) activates the mammalian target of rapamycin (mTOR) which likewise suppresses IRS-1 activation of phosphatidylinositol 3,4,5, triphosphate (PIP3) [14]; ceramides, metabolites of saturated fats known to accumulate in insulin-resistant tissues, have been shown to selectively block the activation of Akt by either promoting the dephosphorylation of active Akt by phosphatase 2A (PP2A) or by blocking Akt translocation to the plasma membrane [15].

In patients with chronic hepatitis C, it has been recently shown that oxidative stress and IR contribute to steatosis, ultimately accelerating the progression of fibrosis [16]. The scope of this brief review is to summarize the current understanding and potential molecular pathways by which HCV contributes to the development of IR, oxidative stress and steatosis.

## 2. HCV interference with insulin signaling

A potential, direct interference of HCV with the insulin signaling cascade was suggested by a study in which liver specimens obtained from 42 non-obese, non-diabetic HCV-infected individuals and 10 non-HCV-infected subjects matched for age and body mass index (BMI) were exposed *ex vivo* to insulin and examined for the contents and phosphorylation status of some insulin-signaling molecules [17]. Insulin-stimulated IRS-1 tyrosine phosphorylation was decreased by 2-fold in HCV-infected patients compared to non-HCV-infected controls, and this was accompanied by decreased p85 PI3-kinase association with IRS-1, resulting in diminished PI3-kinase enzymatic activity and insulin-stimulated Akt phosphorylation [17]. The authors concluded that, in patients with chronic hepatitis C, direct interactions between HCV and insulin signaling components occur, resulting in IR, which may progress to type 2 diabetes in at-risk individuals.

In the transgenic mouse [18], IR associated with the expression of the core-encoding region of HCV could be reversed by treatment with anti-tumor necrosis factor (TNF)-α antibodies. The authors showed that a suppression of tyrosine phosphorylation of IRS-1 may be at least one of the mechanisms by which a high level of TNF-α causes IR in these mice. Thus, in this animal model, the core protein may induce IR indirectly *via* an increased secretion of TNF-α. Nevertheless, *in vitro* models have largely suggested a direct interaction of the core protein with the insulin signaling pathway. An increased proteasomal degradation of IRS-1 and -2 *via* the activation of the suppressor of cytokine signaling (SOCS)-3 has been reported after transient expression of the core protein in hepatoma cells [19]. Genotype-specific mechanisms have been proposed in another *in vitro* study [20], where a downregulation of peroxisome proliferator-activated receptor (PPAR)-γ and an upregulation of SOCS-7 were observed in Huh-7 hepatoma cells transfected with the core protein of genotype 3, whereas the core protein of genotype 1b activated mTOR [20].

Among the indirect mechanisms, increased endoplasmic reticulum (ER) stress has also been reported [21] to lead to the activation of PP2A, an inhibitor of Akt. Activation of PP2A may also dephosphorylate the AMP-activated kinase (AMPK), a key regulator of gluconeogenesis [22]. Thus, PP2A may lead to IR *via* a dual mechanism, i.e. inactivation of the two pivotal kinases Akt and AMPK.

**Figure 2.**
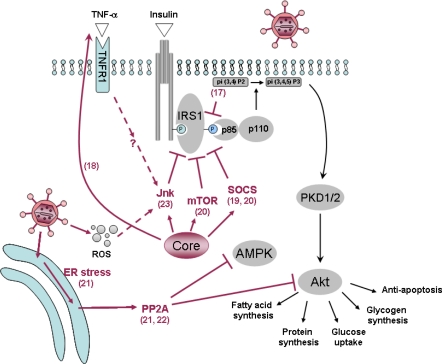
Schematic representation of some of the effects brought about by HCV on insulin signaling in hepatocytes. HCV has been shown to interfere with the insulin pathway at multiple non-exclusive levels: The HCV core can activate inhibitors of insulin signaling including the mammalian target of rapamycin (mTOR) and the suppressor of cytokine signaling (SOCS)-3 and c-Jun N-terminal kinase (JNK), either directly or indirectly via an increased secretion of tumor necrosis factor (TNF)-α, which suppress IRS-1 activation of phosphatidylinositol 3 (PI3)-kinase. Among the indirect mechanisms, an increased endoplasmic reticulum (ER) stress can lead to the activation of the protein phosphatase 2A (PP2A), an inhibitor of Akt. Activation of PP2A may also dephosphorylate the AMP-activated kinase (AMPK), a key regulator of gluconeogenesis. PKD1/2: protein kinase D1/2; p85/p110: subunits p85 and p110 of PI3-kinase. Numbers refer to the bibliographic references.

A potential role of a stress kinase, the c-Jun N-terminal kinase (JNK) has been pointed out in a recent study [23]. The HCV core protein-mediated Ser(312) phosphorylation of IRS-1 was inhibited by a JNK inhibitor in an *in vitro* infection assay using HCV grown in cell culture [23]. The activation of JNK by the HCV core may be direct, but indirect, pro-inflammatory cytokine-mediated mechanisms (*via* an autocrine loop) have not been entirely ruled out. All the information detailed in this section is summarized in Figure 2.

## 3. HCV and oxidative stress

**Figure 3.**
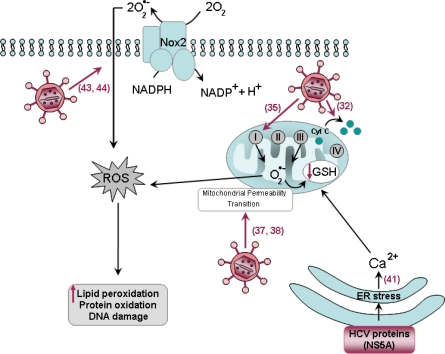
Schematic representation of the effects of HCV on oxidative stress. HCV can induce reactive oxygen species (ROS) via multiple mechanisms: The particular localization of the core protein within the outer membrane of mitochondria may induce increased oxidation of mitochondria glutathione (GSH) and facilitate the uptake of Ca^2+^ into the mitochondria by sensitizing mitochondria to mitochondrial permeability transition. There is an increase in ROS production by mitochondrial electron transport complex I (circles with roman letters, the sites of ROS production in the mitochondrial electron transport chain have been localized in Complex I and Complex III) and a redistribution of cytochrome c (cyt c) from the mitochondrial to cytosolic fractions. The HCV nonstructural proteins including NS5A are associated with the membrane of the endoplasmic reticulum (ER), which activates the release of Ca^2+^ from ER, thereby inducing oxidative stress. NS3 has been shown to trigger ROS production via activation of NADPH oxidase 2 (Nox2). Numbers refer to the bibliographic references.

Oxidative stress is now considered as a key player in the development and the progression of pathogenesis of liver induced by HCV. The following section describes the possible molecular mechanisms involved in increased oxidative stress during hepatitis C (Figure 3).

HCV infection is characterized by increased markers of oxidative stress. For instance, levels of 8-hydroxydeoxyguanosine, a DNA base-modified product generated by reactive oxygen species (ROS) and reactive aldehydes produced by lipid peroxidation, such as 4-Hydroxy-2-nonenal are increased in HCV infection [24, 25]. Under normal conditions, numerous cellular antioxidant systems exist to attenuate oxidant stress and maintain the redox balance of the cell. ROS are cleared from the cell by anti-oxidant enzymes including glutathione (GSH) peroxidase, using reduced gluthatione as substrate. In the context of HCV infection, the levels of gluthatione are significantly decreased [26]. A link between oxidative stress and pathogenesis is also supported by clinical studies suggesting that anti-oxidant therapy could improve liver injury and be helpful in the management of HCV patients [27, 28]. It has been suggested that increased oxidative stress may play an important role in the induction of IR. A significant correlation was observed between homeostatic model assessment (HOMA) and serum levels of thioredoxin, a marker of oxidative stress in chronic hepatitis C patients infected with HCV genotypes 1 or 2 [29].

This increased oxidative stress in hepatitis C may be explained by chronic inflammation. Nevertheless, markers of oxidative stress are also found in HCV carriers with minimal or no liver disease [30] and oxidative damage is also evident before the appearance of histological signs of hepatitis in HCV-transgenic mice [31], indicating that HCV may directly promote oxidative stress in hepatocytes. In addition, it is noteworthy that although both HCV and hepatitis B virus (HBV) cause hepatitis, HCV is particularly more effective at generating oxidative stress [24], suggesting mechanisms that are specific to HCV. Numerous studies have shown that several structural (core) or non-structural (NS3 and NS5A) HCV proteins directly act as inducers of oxidative stress in cell culture [32–34] as well as in transgenic mouse models [32, 35]. The HCV core protein has multiple cellular sites of localization and is largely associated with the ER but it has also been found to localize to the outer membrane of mitochondria via its COOH-terminal region [32, 35, 36]. This particular localization of the protein has been suggested to induce increased oxidation of mitochondria GSH and to facilitate the uptake of Ca^2+^ into the mitochondria [35] by stimulating the Ca^2+^ uniporter activity [37], thus sensitizing mitochondria to mitochondrial permeability transition [37, 38]. In addition, there was an increase in ROS production by mitochondrial electron transport complex I [35] and a redistribution of cytochrome c from the mitochondrial to cytosolic fractions [32].

Besides the core protein, HCV NS5A protein has also been reported to induce oxidative stress. NS5A was shown to significantly increase the ROS levels in Huh-7 hepatoma cells [39]. The HCV nonstructural proteins including NS5A are associated with the membrane of the ER [40]. The association of NS5A with the ER has been suggested to activate the release of calcium from ER stores [41], thereby inducing oxidative stress with parallel activation of Signal Transducers and Activator of Transcription (STAT)-3 and nuclear factor-κB (NF-κB) [39]. This activation of NF-κB and STAT by NS5A may play an important role in inflammation, immune responses, tumor formation and apoptotis [39]. A model of mice transplanted with human hepatocytes to generate chimeric mouse–human livers (SCID/alb-uPA chimeric mice) and infected with HCV, showed that HCV-induced oxidative stress contributes to the activation of pro-apoptotic Bax together with the prevention of the anti-apoptotic BCL-xL, thus sensitizing HCV-infected cells to apoptosis [42].

NS3 has been shown to trigger ROS production via activation of NADPH oxidase 2 (Nox2) in human monocytes from healthy blood donors incubated with recombinant HCV proteins [43] or in mononuclear and polymorphonuclear phagocytes [44]. The latter NS3-activated phagocytes, in turn, induce increased apoptosis of three major subsets of lymphocytes highly relevant in the defense against HCV infection [44].

## 4. Role of the HCV in steatosis development

The reported prevalence of steatosis in patients with chronic hepatitis C varies between 40% and 80%, depending on the features of the population studied in terms of alcohol consumption, prevalence of obesity, diabetes and other risk factors [45]. The prevalence of steatosis in HCV is approximately 2-fold higher than in another common chronic liver disease like hepatitis B [46], suggesting that HCV may directly cause steatosis, at least in some patients. All genotypes are steatogenic, but numerous reports showed that steatosis was more frequent and more severe in patients infected with genotype 3 [47–49]. In this context, it is interesting that especially in patients with genotype 3, the severity of steatosis correlates with the level of HCV replication in liver [47] or in serum [48]. In addition, liver steatosis is significantly reduced or even disappears when patients are successfully treated with antivirals. This effect, again, is more evident in patients with genotype 3, while those with non-3 genotypes may remain steatotic even in case of sustained virological response (SVR) [50, 51]. A relapse after the end of therapy may result in the reappearance of steatosis in patients [52]. *In vitro* studies and a transgenic mouse model have both suggested that the HCV core protein is sufficient to induce a lipid accumulation in hepatocytes [53, 54]. This viral protein is localized on the surface of lipid droplets, and its over-expression seems to further stimulate the formation of lipid droplets. Most models have used genotype 1-derived constructs, but similar results have also been reported using other viral genotypes, including type 3a, which seems to be the most efficient in inducing fat accumulation [55]. In fact, although some degree of intra-hepatocyte lipid accumulation occurs with all viral genotypes, genotype 3 core protein expression induces about 3-fold more fat accumulation than genotype 1 [55], in agreement with the clinical evidence. It has been suggested that a phenylalanine residue at position 164 of the core protein can increase steatosis [56].

**Figure 4.**
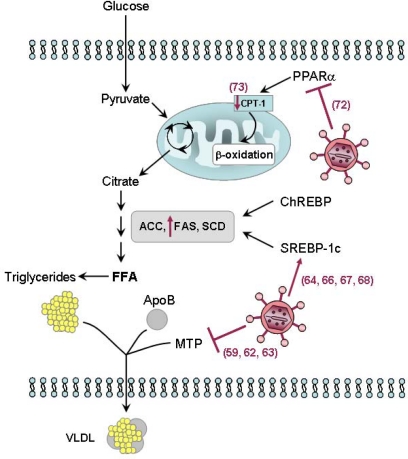
Schematic representation of the effects of HCV on steatosis development. HCV may interfere with lipid metabolism via at least three distinct, non-mutually exclusive mechanisms: (i) Impaired secretion. HCV may interfere with the very-low density lipoprotein (VLDL) assembly and/or secretion. Both apolipoprotein B (ApoB) secretion and microsomal triglyceride transfer protein (MTP) activity are impaired by HCV core protein expression. (ii) Increased *de novo* synthesis of free fatty acids (FFA). HCV has been reported to upregulate sterol regulatory element binding protein (SREBP)-1c signaling pathway, leading to the up-regulation of enzymes involved in lipogenesis such as FA synthase (FAS). (iii) Impaired FA degradation. The HCV core protein reduces the expression of peroxisome proliferators-activated receptor (PPAR)-α, a nuclear receptor regulating several genes responsible for FA degradation, as well as that of mitochondrial carnitine palmitoyltransferase type 1 (CPT)-1, the rate-limiting enzyme of mitochondrial β-oxidation. ACC: acetyl-CoA carboxylase; SCD: stearoyl coenzymeA desaturase. Numbers refer to the bibliographic references.

HCV may interfere with lipid metabolism via at least three distinct, non-mutually exclusive mechanisms (Figure 4): impaired secretion, increased *de novo* synthesis, and/or impaired degradation.

Impaired secretion of lipids from the infected hepatocyte has been the first proposed mechanism of HCV-induced steatosis. In fact, serum levels of apolipoprotein B (ApoB) and cholesterol are reduced in chronic hepatitis C patients [57, 58], suggesting that HCV may interfere with very-low density lipoprotein (VLDL) assembly and/or secretion. The disappearance of fatty liver in sustained virological responders to antiviral therapy correlates with normalization of ApoB levels [51, 57]. Thus, clinical data suggest that HCV may interfere with VLDL secretion, a defect corrected by antiviral treatment.

Transgenic mice have been developed that express either the HCV core protein (CoreTg), ApoAII (ApoAIITg) or both genes (CoreTg/ApoAIITg). Based on this experimental model, it has been shown that core-expressing mice have impaired VLDL-triglyceride and Apo-B secretion that can be rescued by hepatic expression of ApoAII [59]. In HCV core protein-expressing mice, VLDL particle size and abundance was markedly reduced compared with non-transgenic mice and the CoreTg/ApoAIITg mice. The CoreTg mice also exhibited a significant decrease in microsomal triglyceride transfer protein (MTP) activity compared to non-transgenic mice [59].

MTP is a heterodimeric lipid transfer protein present in the luminal side of the ER in the liver, intestine and heart (for review see [60]). It plays a key, rate-limiting role in the assembly and secretion of VLDL by lipidating nascent ApoB to form the mature ApoB lipoprotein [61]. Thus, the consequence of its inhibition is the accumulation of triglycerides within the cytoplasm of the cell, i.e. steatosis. Data in human liver are in agreement with this proposed mechanism, since the intrahepatic levels of MTP mRNA is reduced in patients with chronic hepatitis C [62] and since a common polymorphism in the promoter region of the MTP gene associates with a higher degree of hepatic steatosis in HCV patients [63].

The association between HCV and intrahepatic oxidative stress has been discussed in the above section. The production of ROS may also result, among other effects, in the peroxidation of membrane lipids and structural proteins, such as those involved in the trafficking and secretion apparatuses. This would then block VLDL secretion, leading to steatosis.

HCV may also induce steatosis *via de novo* synthesis of FFA. In this context, HCV has been reported to upregulate the SREBP-1c signaling pathway [64]. Recent studies have shown that HCV infection increases the proteolytic processing of SREBP precursors in hepatic cells [65] and that HCV NS2 and NS4B proteins can up-regulated SREBP-1c at the transcriptional level [66, 67]. Interestingly, NS4B-induced SREBP activation requires the activation of the Akt signaling pathway [67]. Consequently, the promoter activity of FAS, one target gene of SREBP-1c, is up-regulated upon expression of NS2 [66], NS4B [67] as well as HCV core [68] proteins. Recent confirmation of the up-regulation of FAS by HCV was provided in the *in vitro* infectious system [69].

Chimpanzees experimentally infected with HCV show an increased intrahepatic activity of enzymes involved in lipogenesis, such as ATP citrate lyase, which are also regulated by SREBP-1c [64]. The HCV core protein may additionally bind to and activate the DNA-binding domain of the retinoid receptor a (RxRa), a transcriptional regulator that controls many cellular functions, including cellular lipid synthesis [70]. On the other hand, accumulation of fat in hepatocytes transiently expressing the HCV core protein seems to depend on the presence of exogenous lipids, which indirectly decreases the likelihood of a significant FA neosynthesis activated by this viral protein [55]. However, one cannot rule out that other viral proteins may activate the neosynthesis of FA.

HCV may finally cause steatosis by impairing FA degradation. Transfection of hepatoma cells with the HCV core protein is followed by a reduced expression of PPARα, a nuclear receptor regulating several genes responsible for FA degradation [71]. These same authors have also reported a down-regulation of mitochondrial CPT-1, the rate-limiting enzyme of mitochondrial β-oxidation, which is the main catabolic pathway of FAs [71]. A reduced expression of CPT-1 in the liver of chronic hepatitis C patients has been reported [72]. As the regulation of several genes, such as CPT-1, is transcriptionally controlled by PPARα, it is possible that the reported effects may be secondary to PPARα down-regulation. PPARα mRNA is significantly reduced in the liver of HCV-infected patients [73], with a predominance in genotype 3 compared to genotype 1 [74]. Overall, the data support the hypothesis that the HCV core protein may modulate the expression of various lipid degradation-associated genes, possibly *via* the down-regulation of PPARα.

## 5. Clinical consequences

The clinical impact of steatosis, oxidative stress and IR in chronic hepatitis C patients includes accelerated liver disease progression and reduced response to interferon-α (IFN-α)-based therapy. Antioxidants have been largely used in the past to treat chronic hepatitis C patients, either when antiviral therapy was contraindicated or had failed, or in addition to IFN-α-based therapy. The results are quite controversial, and show, in the best scenario, a reduction of liver enzyme levels without significant changes of the HCV viral load [28, 75].

The impact of virally-induced steatosis on response to therapy is unclear. When the chances of SVR have been evaluated in clinical trials, virtually all studies show that steatosis is an independent factor of poor response only in genotype non-3-infected patients but not in those with genotype 3 (reviewed in [76]), suggesting that viral steatosis may not reduce the response to IFN-α. Similarly, steatosis due to HCV is not independently associated with the fibrosis stage, whereas metabolic steatosis is [77]. Again, if the multivariate logistic regression analysis includes IR, then the effect of metabolic steatosis disappears, replaced by IR [78]. There is, in addition, a wide consensus on the impact of IR on SVR, although it is not evident to dissect the effect of viral IR, if any, from that due to host metabolic factors, essentially due to the lack of viral markers predictive of virally-induced IR. IR reduces the response to treatment and is indeed a better predictor of SVR than metabolic steatosis [79]. Increasing levels of IR are associated with reduced rates of initial virological response [80–82] and SVR, independently of the HCV genotype [79, 83–87]. Why IR should reduce the response to IFN-α is unclear, but some investigators have pointed out that deregulation of SOCS-3 may be involved in both IR and lack of response to therapy [88].

Thus, it seems as if only the non-3a-associated steatosis, presumably metabolic, is decreasing SVR and accelerating fibrosis progression, suggesting that it is not steatosis *per se* that matters, but its pathogenesis. Most data converge to support the notion that IR is a major factor with clinical consequences in chronic hepatitis C, both at the level of liver fibrosis progression and during therapy with IFN-α-based regimens.

Thus, correcting IR in chronic hepatitis C patients has become a priority, although current measures are empirical rather than pathogenesis-driven. A short, three-month program of weight loss in 19 chronic hepatitis C patients was able to reduce liver enzymes and improve fibrosis, despite no effects on viremia [89], but the impact on response to therapy was not evaluated. More recent trials using insulin sensitizers, like pioglitazone and metformin, have attempted to increase the rate of early and sustained response to therapy. Results, however, are negative [90, 91] or inconclusive [92, 93], clearly warranting further trials with alternative schedules.

## 6. Conclusions and relevant questions for the future

Our comprehension of the molecular mechanisms by which HCV induces IR, oxidative stress and steatosis has evolved significantly. A growing body of evidence indicates that these disorders are closely related to the progression of liver damage in HCV-infected patients, even though a complete picture of the molecular pathways leading to their interconnection has not yet been established. From the patient management point of view, these effects on hepatitis C progression warrant specific and effective measures to correct such anomalies. In this context, some aspects need to be addressed by future studies:

- What is the exact relationship between virally-induced steatosis and HCV replication? Can we pharmacologically manipulate the lipid metabolism to inhibit HCV replication in vivo?
- What is the long-term clinical impact of purely viral IR? Is this associated with an increased cardiovascular risk, as is the metabolic syndrome-associated IR?
- Are life-style changes – especially increased physical activity – associated with metabolic changes in the liver, and, if this is the case, what would be the consequences for HCV replication, HCV-related steatosis and HCV-related IR?
